# Costs attributable to hypercholesterolemia in a single period and over the life cycle

**DOI:** 10.1007/s10198-024-01684-0

**Published:** 2024-03-22

**Authors:** Stephanie Reitzinger, Miriam Reiss, Thomas Czypionka

**Affiliations:** 1 Institute for Advanced Studies, Josefstädter Str. 39, 1080 Vienna, Austria; 2 London School of Economics and Political Science, London, UK

**Keywords:** Hypercholesterolemia, Cardiovascular diseases, Societal costs, Macrolevel data, Lifecycle, Austria, C82, I10, I18

## Abstract

Hypercholesterolemia is a major risk factor for atherosclerotic cardiovascular disease leading to reduced (healthy) life years. The aim of this study is to quantify the societal costs associated with hypercholesterolemia. We use epidemiologic data on the distribution of cholesterol levels as well as data on relative risks regarding ischemic heart disease, stroke, and other cardiovascular diseases. The analytical approach is based on the use of population-attributable fractions applied to direct medical, direct non-medical and indirect costs using data of Austria. Within a life-cycle analysis we sum up the costs of hypercholesterolemia for the population of 2019 and, thus, consider future morbidity and mortality effects on this population. Epidemiologic data suggest that approximately half of Austria’s population have low-density lipoprotein cholesterol (LDL-C) levels above the target levels (i.e., are exposed to increased risk). We estimate that 8.2% of deaths are attributable to hypercholesterolemia. Total costs amount to about 0.33% of GDP in the single-period view. In the life-cycle perspective, total costs amount to €806.06 million, €312.1 million of which are medical costs, and about €494 million arise due to production loss associated with hypercholesterolemia. The study points out that significant shares of deaths, entries into disability pension and care allowance, full-time equivalents lost to the labor market as well as monetary costs for the health system and the society could be avoided if LDL-C-levels of the population were reduced.

**Supplementary Information:**

The online version contains supplementary material available at 10.1007/s10198-024-01684-0.

## Introduction

High levels of non-high-density lipoprotein cholesterol (non-HDL-C), which include high levels of low-density lipoprotein cholesterol (LDL-C), referred to as *hypercholesterolemia*, are a major risk factor for cardiovascular diseases and are thus associated with a loss of life and quality of life [1, 2]. Hypercholesterolemia can be genetically determined (familial hypercholesterolemia) or––more commonly––a consequence of diabetes mellitus, an underactive thyroid, obesity or an unhealthy lifestyle [3]. Elevated non-HDL-C was named as the 8th leading risk factor for death in 2019 [3] and is estimated to be responsible for 3.9 million deaths globally in 2017 [4].

Despite this high relevance, there is only limited evidence regarding the economic burden of hypercholesterolemia in European countries. The existing evidence indicates that cardiovascular diseases caused by elevated cholesterol levels bring about considerable costs to society, as those affected require consistent medical treatment and may become incapacitated for work [5–7].

The objective of this study is to estimate the cost-of-illness of hypercholesterolemia in the Austrian case. We specifically investigate what costs could be avoided if the entire Austrian population never exceeded their respective cholesterol target values over their lifetime, i.e., were not subject to increased risk for cardiovascular disease due to hypercholesterolemia. We take into account variation in target values for high-risk and very-high-risk groups as well as for familial hypercholesterolemia. The cost analysis, furthermore, includes a life-cycle model that allows for consideration of cumulative effects following the model’s structure in Murphy and Topel [8]. In this model, we estimate the costs for cardiovascular diseases over the expected lifespan of the current population resulting from the distribution of hypercholesterolemia-associated risks in 2019.

## Methods

We used a prevalence-based approach calculating population-attributable fractions for different cost categories (direct medical, direct non-medical, and indirect costs). Prevalence data were combined with relative risk estimates of hypercholesterolemia-related diseases. The base year of our analysis was 2019. The estimation of costs was conducted on a basis of 5-year age groups separately for men and women. We calculated cost of illness as the difference in expenditures between the status-quo population and a hypothetical population in which everyone achieves their target cholesterol levels. In a complementary life-cycle analysis, we estimated cumulative costs of hypercholesterolemia for the population of 2019 considering future mortality and morbidity effects. In our cost analysis, we aimed to incorporate direct and indirect costs, as well as public transfers for sick pay and disability pensions in association with hypercholesterolemia incurred to the society.

### Definition of hypercholesterolemia and risk groups

The cost evaluation in our study was based on risk data referring to total cholesterol, as no adequate data referring to LDL-C were available. Since the (non-)achievement of the target for LDL-C is closely related to the (non-)achievement of the target for total cholesterol [9], we approximated that an elevated risk starts at 60% of total cholesterol in accordance with ESC Guidelines [10]. Accordingly, hypercholesterolemia is defined as values above 5 mmol/l total cholesterol for persons at low or moderate risk, above 3 mmol/l for persons at high risk and above 2.3 mmol/l for persons at very high risk.

In accordance with ESC Guidelines [10], the definition of *very high risk* is predominantly determined by pre-existence of cardiovascular diseases. Very-high-risk-persons show the following characteristics: documented myocardial infarction, documented unstable angina, documented stable angina, documented coronary revascularization, documented stroke and TIA, documented peripheral arterial disease, ASCVD unequivocal on imaging known to be predictive of clinical events, such as significant plaque on coronary angiography or CT scan or on carotid ultrasound, diabetes mellitus with target organ damage or at least three major risk factors, or early onset of type-1-diabetes of long duration (> 20 years), severe chronic kidney disease, a calculated score >= 10% for 10-year risk of fatal CVD, or familial hypercholesterolemia with ASCVD or with another major risk factor. *High risk* is mainly defined by total cholesterol values above 8 mmol/l, a risk score above 5%, pre-existing diabetes mellitus for at least 10 years or familial hypocholesterolemia. In addition to these factors, the risk score also considers smoking status, systolic blood pressure, and the geographic region. A detailed definition is provided in the Online Resources (S1).

### Distribution of total cholesterol levels in the population

We defined nine cholesterol categories where each category is associated with a higher risk for cardiovascular diseases relative to the base category. The base category includes total cholesterol levels up to the target value, and categories increase by 1 mmol/l up to the ninth category.

To determine the distribution of the Austrian population over the defined cholesterol categories, we had to draw on German means and standard deviations found by Scheidt-Nave et al. [11]. Their findings were derived from interview and laboratory data in the German Health Interview and Examination Survey for Adults 2008–2011. Based on findings of the NCD Risk Factor Collaboration [12], it can be assumed that the Austrian and the German population are similar regarding total cholesterol levels. To obtain a distribution, stratified by age and sex, which corresponds to our defined cholesterol categories, we, further assumed that the distribution of total cholesterol levels in the population can be approximated by a normal distribution based on observational data [11, 13]. Finally, we validated the resulting estimated prevalence of hypercholesterolemia for Austria’s adult population, as outlined in the Online Resources (S3).

### Prevalence of very high and high risk for hypercholesterolemia-associated cardiovascular diseases in the population

To account for the different target values for persons who are at high or very high risk for cardiovascular diseases in our model, we deduced their prevalence using multiple data sources.

The prevalence of very high risk was determined as follows. In a first step, we used the prevalence of coronary heart disease (ICD 10: I.20–I.25) [14] and stroke (ICD 10: I.64) as a proxy for the prevalence of the very-high-risk population in Austria [15]; we deducted 10% of the combined prevalence to adjust for comorbidity of coronary heart disease and stroke [16] In a second step, we derived the distribution of this group over cholesterol groups from results of a German study based on a cohort of patients in statin treatment [Table 3 in 17]. 18.5% of the very-high risk patients achieved the target of < 3 mmol/l for 2016 and around half thereof achieved the target of < 2.3 mmol /l for the year 2019 [18]. We adjusted the percentage of the cholesterol groups who do not reach the target cholesterol level to those used in the present study.

The prevalence of high risk was determined as follows. Around 60% of patients on statin treatment have an established cardiovascular disease; two thirds of these are heart diseases and/or stroke [9, 18]. We used this information as a proxy for the number of high-risk persons with total cholesterol levels between 3 and 8 mmol/l: first, we calculated the total sum of high- and very-high-risk-patients by dividing the absolute number of very-high-risk patients by 0.4; secondly, given the total sum of high- and very-high-risk-patients and the total sum of very-high-risk-patients we could deduce the sum of high-risk patients. For the population under 45 years of age, we used an adapted approach to derive the prevalence of high risk of cardiovascular disease. First, we used the proportion of persons with > 8 mmol/l total cholesterol. Second, we included persons who suffer from familial hypercholesterolemia, which is estimated to be approx. 0.33% of the general population [19]. Third, we included persons under the age of 45 who suffer from diabetes mellitus. According to the 2019 Austrian Health Interview Survey, diabetes mellitus occurs in 0.4% of 15–29-year-old women, 1.3% of 30–44-year-old women and 4% of 45–54-year-old women. For men, it is 0.6% of 15–29-year-olds, 0.9% of 30–44-year-olds and 6.7% of 45–54-year-olds [20]. Men over 40 years of age who, in addition to the presence of hypercholesterolemia, also smoke and have elevated blood pressure, would by definition also belong to the high-risk group, but are absent from our high-risk prevalence due to missing data in this regard. Due to missing data with respect to high-risk persons under the age of 45 years, furthermore, we assumed an equal distribution of prevalence across cholesterol groups.

### Relative risks for cardiovascular diseases

We conducted a review of the epidemiologic literature regarding hypercholesterolemia-associated risks for morbidity and mortality regarding cardiovascular diseases. The approach used in the review is described in the Online Resources (S2). We eventually drew relative-risk estimates from a comprehensive epidemiologic study by the Prospective Studies Collaboration (PSC) [21]. The PSC evaluated data from 61 long-term studies from mostly European countries and North America covering around 900,000 individuals. The study found associations between total cholesterol and death from ischemic heart disease, stroke, and other pooled diseases of the cardiovascular system after adjusting for age, sex, systolic blood pressure, BMI, smoking status, and the presence of diabetes. The study reports hazard ratios with respect to a decrease of total cholesterol by 1 mmol/l by sex and age groups. We use the reciprocal, i.e., the increase of risk with an increase of total cholesterol by 1 mmol/l, as an approximation for relative risks.

Table 1 summarizes the mean hazard ratios (and confidence intervals) per 1 mmol/l higher total cholesterol for different age groups regarding ischemic heart disease, stroke, and other cardiovascular diseases. In the general population without any relevant prior diseases, the elevated risk starts at 5 mmol/l of total cholesterol [10]. We can see in the literature that the relationship between total cholesterol level and risk is approximately log-linear at different rates with respect to age [21]

**Table 1:** Hazard ratios for death from ischemic heart disease, stroke, and other cardiovascular diseases, by age groups per 1 mmol/l total cholesterol

Age	Ischemic heart disease	Stroke	Other cardiovascular diseases
40–49	2.22 (2.13–2.38)		1.61 (1.45–1.82)
50–59	1.75 (1.72–1.82)	1.10 (1.03–1.18)	1.33 (1.27–1.41)
60–69	1.47 (1.45–1.52)	1.08 (1.03–1.12)	1.20 (1.16–1.25)
70–79	1.27 (1.23–1.28)		1.12 (1.09–1.18)
80–89	1.18 (1.12–1.22)		

Based on hazard ratios found by the Prospective Studies Collaboration [21]

### Direct medical costs

For the calculation of medical costs, we used the costs of cardiovascular diseases (i.e., ischemic heart disease, stroke, other cardiovascular diseases) by sex and 5-year age groups. Since data on cost accounts by disease are not collected in Austria, we used the cost shares by age and sex from the most recent German medical cost account of the year 2015 [22] and applied them to total Austrian health expenditures (without long-term care) by age groups and sex. We derived the Austrian age-specific health expenditure data from the distribution of public health expenditures in the Austrian intramural (i.e., hospitals) and extramural (i.e., physician practices) health sectors from data provided by the Austrian Ministry of Social Affairs, Health, Care and Consumer Protection, the Austrian Federation of Social Insurances, the Austrian Health Insurance Fund and the Austrian Private Hospital Financing Fund [23, 24]. Because these expenditure data covered approx. 75% of Austria’s total current health expenditure [25], we approximated additional private health expenses using a factor of 1.33 (= 1/0.75) for each age group. Data on public and private health expenditures, thus, comprise expenses on diagnosis, treatment, prevention, therapeutic procedures (e.g., speech and occupational therapy), medication, rehabilitation as well as any related resource consumption (e.g., administrative costs).

### Indirect costs

Indirect costs arise due to production losses because of sick leave, disability, or premature death associated to hypercholesterolemia. For the calculation of indirect costs, we used diagnosis-specific data on sick leaves, entries into disability pension and deaths as well as data on labor-force participation rates and total wage costs (including employees’ and employers’ social security contributions and payroll taxes) all by sex and age groups. Details on this data are provided in the Online Resources (S5).

### Long-term care and public transfers

Further hypercholesterolemia-associated public expenditures include care allowances (as a proxy for expenditure long-term care expenditure), sickness benefits, and disability pensions. We received diagnosis-specific data on new entries into each of these categories for the year 2019 from the Austrian Social Health Insurance and derived the shares of entries attributable to hypercholesterolemia by sex and age group. More details on the underlying calculations can be found in the Online Resources (S5).

### Population-attributable fractions

Data on prevalence and relative risks for ischemic heart disease, stroke, and other cardiovascular diseases were combined to determine population-attributable fractions (PAF) for each considered disease by sex and age group [26]. We divided the population into 8 groups according to their level of total cholesterol (2.3–2.9 mmol/l, 3–3.9 mmol/l, 4–4.9,,…, more than 8.9 mmol/l)

$${\text{PAF}}\left(a,d\right)=\frac{{p}^{0}(a)+(\sum_{l=1}^{8}{p}^{l}(a)*{{\text{RR}}}^{l}\left(a,d\right))-1}{{p}^{0}(a)+(\sum_{l=1}^{8}{p}^{l}(a)*{{\text{RR}}}^{l}\left(a,d\right))}$$

*a*: age, *d*: disease, *p*: prevalence, *l*: cholesterol group l = {1,…8}, RR: relative risk

The resulting disease- and age-specific population-attributable fractions––with respect to each of the eight cholesterol groups––were then applied to the relevant determinants of costs mentioned above (i.e., health expenditure, care allowances, attendance rate, and deaths).

### The life-cycle model

Using population-attributable fractions, the costs of hypercholesterolemia can be calculated for Austria’s population in 2019 by assuming the entire population achieved their target cholesterol values and had never previously exceeded them. In addition to this one-period view, we furthermore estimated the sum of future costs of the current population tracking them until the end of their life [8]. For this life-cycle model, survival probabilities of each age group were required in addition to population-attributable fractions.

The survival probability is the probability of surviving the period from age *a* to age *t*, given survival to age *a*. To estimate the survival probability for the hypothetical population, the hypercholesterolemia-non-attributable fractions were multiplied by the observed sex-age-disease-specific death rates $\lambda$ [27] and added up over all disease groups *d* within an age group *a*. The mortality probability of the hypothetical population, which corresponds to the counter-probability of the survival probability, is given by:

$${\lambda }^{N}\left(a\right)= \sum_{d\in \{{\text{ICD}}\} }\lambda \left(a,d\right) \left(1-{\text{PAF}}\left(a,d\right)\right)$$

We weighted future cash flows with the probability of survival for each transition of a birth cohort to the next age group (i.e., the time horizon of the model varies with age with a maximum of about 92 years for those born in 2019). In addition, we discounted future costs and transfers at a rate of 3%. For better interpretation of the results of the life-cycle model, we furthermore calculated the annuity of the present values assuming a period of 92 years.

### Sensitivity analysis

To examine the sensitivity of our model with respect to the discount rate, we increased (I) and decreased (II) the discount rate by one percentage point. Furthermore, we used the upper value (III) and the lower value (IV) of the 95% confidence interval for relative risks.

## Results

### Prevalence

**Fig. 1:**
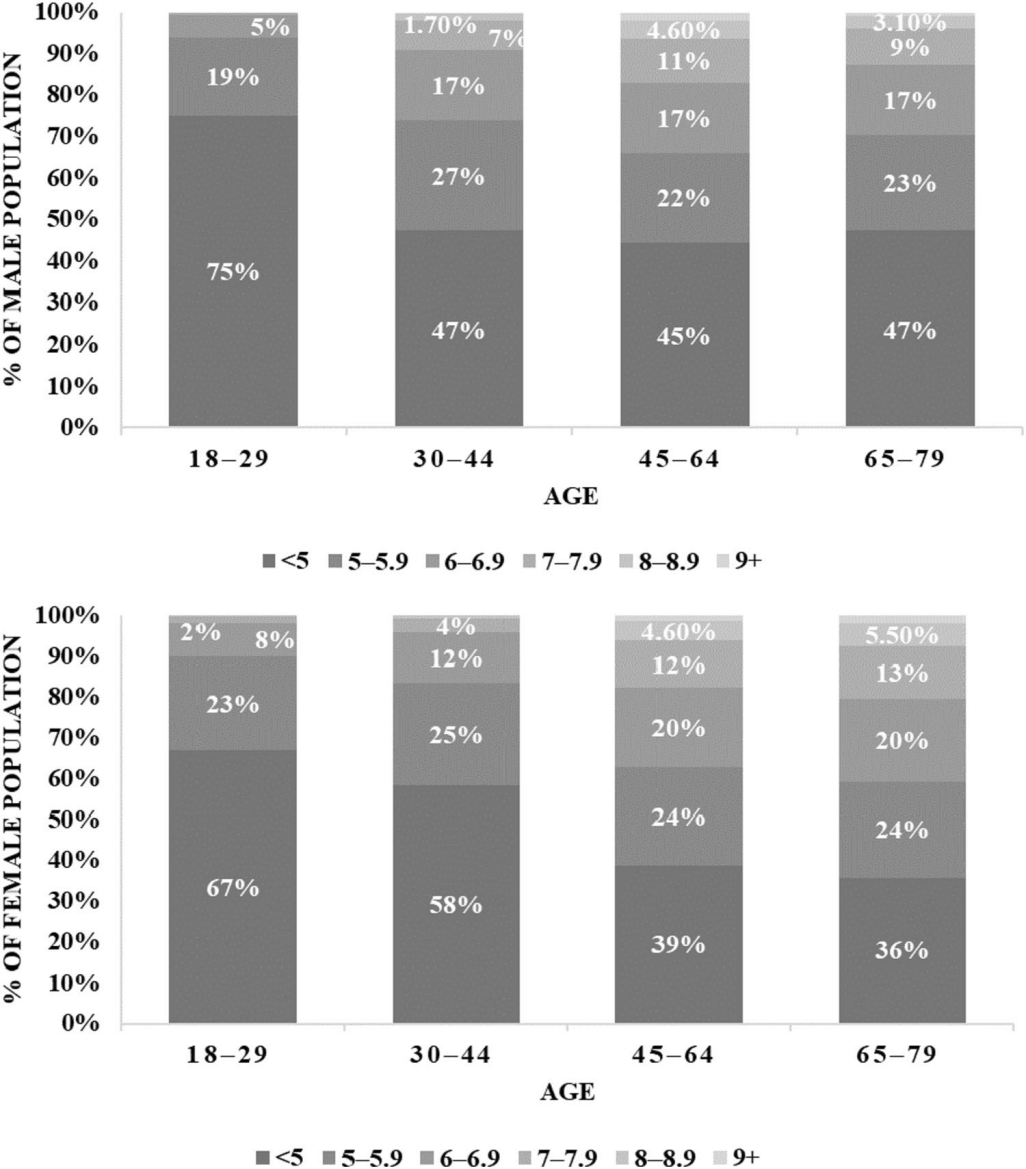
Distribution of total cholesterol in mmol/l by age categories (top: men; bottom: women); the distribution is derived from data by Scheidt-Nave et al. [11]

Figure 1 illustrates the estimated distribution of total cholesterol levels for men and women (right and left, respectively). 55% of men and 61% of women in the age group 45–64 and 53% of men and 64% of women in the age group 65–79 exhibit total cholesterol levels above 5 mmol/l. Thus, elevated total cholesterol levels are more common among women than men. This can partly be explained by the fact that men are more often exposed to high or very high risk for cardiovascular diseases and thus undergo a cholesterol-lowering therapy. Many patients on statin treatment, however, do not achieve their LDL-C target value and remain at high or very high risk for cardiovascular diseases [9, 17].

We estimated that a total of 1.15 million persons (15.2% of the population over 15 years) were at high or very high risk for hypercholesterolemia-associated cardiovascular diseases in Austria in 2019. Moreover, a total of 360,000 persons were at very high risk. A table of prevalence by age groups is provided in the Online Resources S4.

### Deaths

We estimated that 6844 deaths in the year 2019 were attributable to hypercholesterolemia. This corresponds to 8.2% of total deaths or 28.6% of deaths from cardiovascular diseases (under the age of 90) [28]. 54% of these deaths were men. This translates into reduced life expectancy: life expectancy at birth would increase by 0.6 years for women and by about 1 year for men if the entire population achieved their target cholesterol levels.

### Direct medical costs

Hypercholesterolemia-associated medical costs across all age groups and cardiovascular diseases amount to a total of €834.7 million or 2.35% of current health expenditure (excluding expenditures for long-term care) in 2019 [25] (see Table 2). About 40% of total medical costs arise in hospitals (i.e., intramural sector) and 29% in physician practices (i.e., extramural sector).

**Table 2:** Costs of hypercholesterolemia in Austria in the 1-year-model and life-cycle model with the base year 2019

	Life-cycle annuity	1-year-model	
	in € million	in € million	in % of 2019 base value^*^
Direct medical costs	312.06	834.7	2.35%^a^
Intramural	104.38	340.2	
Extramural	113.87	239.7	
Others	93.81	254.8	
Direct non-medical costs	42.4	28.3	
Sick pay	16.6	20.3	2.27%^b^
Care allowances	0.01	− 3.4	− 0.13%^c^
Disability pension	25.8	11.5	0.16%^d^
Indirect costs	494.0	303.2^f^	0.09%^e^
Total costs	848.46	1,166.2	0.34%^e^

^*^Each cost category is referred to a base value that has been chosen as the most adequate reference for the respective category

^a^Current health expenditures excluding long-term care [25]

^b^Total sick pay expenses (referring to data from the Austrian Health Insurance Fund)

^c^Total expenses of care allowances [28]

^d^Total expenses of disability pension [28]

^e^GDP [28]

^f^The value of production loss excludes future loss of wages in the 1-year-model

The life-cycle model cumulates total medical costs of the population in 2019 until their death, discounted at an interest rate of 3%. The model yielded an annuity of hypercholesterolemia-attributable medical costs of €312.06 million. Although the life-cycle model accounts for lower life expectancy of persons with hypercholesterolemia and, therefore, saved medical costs in older ages to treat other diseases, the annuity of health expenditure associated with hypercholesterolemia is substantial.

### Indirect costs

We find that because of sick leaves, deaths, and disability entitlements attributable to hypercholesterolemia, 3928 full-time equivalents were lost to the Austrian labor market in 2019. The associated production loss amounts to €303.2 million or 0.09% of the gross domestic product of 2019 [28] according to the one-period analysis (not considering future loss of wages). The life-cycle model additionally accounts for future production losses due to permanent inability to work and yielded, therefore, higher indirect costs of €494.0 million per year due to hypercholesterolemia.

### Long-term care and public transfers

In the one-period analysis, we estimated that a total of €20.3 million in sickness benefits for hypercholesterolemia-associated diseases were paid out in Austria in 2019. This corresponds to 2.27% of total expenditure for sickness benefits. The life-cycle model, which also considers sickness benefits for other diseases with increasing age, yielded annual expenditures due to hypercholesterolemia of €16.6 million.

In the context of long-term care, the one-period analysis resulted in savings due to hypercholesterolemia as decreased mortality in the hypothetical population leads to higher expenses for care allowances. The savings amount to €3.4 million for the year 2019. In the life-cycle model, this result is reversed with a positive amount of €0.01 million. The reason for this is that the life-cycle model takes into account the cumulative effect of care allowances to be paid in future: new entries into long-term care allowance due to hypercholesterolemia on average occur in lower age groups than new entries due to other diseases.

Regarding disability pensions, the one-period analysis resulted in transfers attributable to hypercholesterolemia of €11.5 million in the year 2019. This corresponds to 0.16% of total expenditure for disability pension benefits and concerns 4.6% of new disability entitlements in 2019. In the life-cycle model, the annual sum of transfers is again higher than in the one-period model because cumulative expenditures for disability pensions over the life cycle are included. The resulting annuity amounts to €25.8 million.

### Sensitivity analysis

Table 3 presents the results of the sensitivity analysis using variations in the discount rate (2% and 4%) and relative risks (upper and lower bound of the 95% confidence interval). Based on these four parameter changes, the annuity of the medical costs ranges between €209.28 and €593.13 million, the annuity of sickness benefits between €15.5 and €20.5 million, the annuity of the care allowance between €-0.22 and €0.28 million, the annuity of disability pensions between €20.4 and €28.8 million, and the annuity of indirect costs between €570.1 and €692.9 million.

**Table 3:** Sensitivity analysis (annuities in € million)

		Medical costs	Sick pay	Care allowance	Disability pension	Indirect costs
Base model		312.1	16.6	0.01	25.8	494.0
Discount rate	2%	209.3	15.5	0.00	25.9	486.8
	4%	396.0	17.7	0.03	25.6	497.3
95%-CI of relative risks^a^	Lower bound	387.4	16.1	− 0.22	20.4	454.0
	Upper bound	593.1	20.5	0.28	28.8	524.7

^a^Data from [21]

## Discussion

Around half of Austria’s population are at increased risk of cardiovascular disease due to elevated levels of blood cholesterol. In 2019, we estimated that hypercholesterolemia was associated with 8.2% of deaths. According to the one-period analysis, the Austrian society was burdened with costs of €1.14 billion due to hypercholesterolemia. Since the treatment of cardiovascular diseases is resource intensive, the majority of these costs arose in the health system with direct medical costs of €834.7 million.

Our life-cycle model views costs from a different angle as it incorporates future costs arising over the life cycle of individuals and thus beyond the period under investigation. According to this model, total costs amount to approx. €806.06 million per year. In contrast to the one-period analysis, the majority of costs over the life cycle result from production losses as individuals fall ill and become (permanently) unable to work. Although medical costs of hypercholesterolemia over the life cycle are partly offset by costs that would be caused by other diseases later in life, our results point to a substantial saving potential in the health system by lowering cholesterol in the population. Furthermore, we conclude from our sensitivity analysis that these results are robust to the use of varying discount rates and relative risks.

Our research contributes to existing knowledge regarding costs of hypercholesterolemia and adds results from a societal and longitudinal perspective. While previous research has mostly focused on medical costs associated with hypercholesterolemia and the potential of therapy (adherence) [5, 29], our study adds novel insights about the economic and, eventually, societal costs of hypercholesterolemia.

In contrast to a cross-sectional analysis, our life-cycle model allows for a better representation of cumulative effects that occur beyond the period under investigation. It does, however, involve a higher level of uncertainty as it relies on assumptions regarding, e.g., interest rates. Furthermore, despite taking a longitudinal perspective, the life-cycle model is based on cross-sectional data; hence, all model parameters, e.g., labor-force participation rates, distribution of total cholesterol over age groups, etc., are assumed to remain constant over time. Both approaches therefore come with certain benefits and drawbacks.

After all, our study might underestimate the true costs of hypercholesterolemia for several reasons. In the context of long-term care, true costs are likely underestimated as care allowances by design only cover a part of actual expenses for long-term care. In particular, the burden of informal care is underrepresented. Moreover, because diagnostic information is only recorded for first-time recipients of care allowance, we do not have data on increases in care allowances as a consequence of developing cardiovascular diseases at a later point of time.

Further limitations of the study include the use of hazard ratios as an approximation of relative risks, the use of data on mortality risks as an approximation for morbidity risks, and the use of total cholesterol rather than LDL-C values. We had to approximate the Austrian distribution of total cholesterol levels within age groups using data from Germany which we believe to be largely representative, but still may not be entirely accurate for the Austrian case. For our estimate of the prevalence of persons at a (very) high risk of cardiovascular disease due to hypercholesterolemia, we made conservative assumptions (i.e., for which we had evidence) because they underlie some degree of uncertainty. Considering this, we believe that our estimates may be underestimating actual expenses.

Despite its limitations, our study represents a valuable addition to the few existing studies aiming to quantify the costs associated with hypercholesterolemia. We believe Austria is an interesting case since the results of our study might be comparable to other European countries showing similar prevalence of hypercholesterolemia under similar health systems.

## Conclusion

We conclude from our analysis that lowering the prevalence of hypercholesterolemia in the population will generate benefits for the society, both in the short and in the long term. We underpin former results [5, 29] suggesting that the provision of therapies and initiatives to enhance therapy adherence could reduce a large share of cardiovascular deaths, and, additionally, argue that high societal costs could be avoided.

## Supplementary Information

Below is the link to the electronic supplementary material.

Supplementary file1 (DOCX 61 KB)

## Data Availability

Data and materials are available on request.
