# Supplementary material for: Costs attributable to hypercholesterolemia in a single period and over the life cycle
Source: Eur J Health Econ. 2024 Mar 22;25(9):1595–603. doi: 10.1007/s10198-024-01684-0 (PMC11512912; doi:10.1007/s10198-024-01684-0)
Supplement: Supplementary file 1 — Supplementary file1 (DOCX 61 KB) [file 10198_2024_1684_MOESM1_ESM.docx]

***S1 - Definition of high and very high risk of cardiovascular disease according to ESC Guidelines [1]***

| Very high risk | High risk |
| --- | --- |
| People with any of the following:   - Documented ASCVD, either clinical or unequivocal on imaging. Documented ASCVD includes previous ACS (MI or unstable angina), stable angina, coronary revascularization (PCI, CABG, and other arterial revascularization procedures), stroke and TIA, and peripheral arterial disease. Unequivocally documented ASCVD on imaging includes those findings that are known to be predictive of clinical events, such as significant plaque on coronary angiography or CT scan (multivessel coronary disease with two major epicardial arteries having >50% stenosis), or on carotid ultrasound. - DM with target organ damage^a^, or at least three major risk factors, or early onset of T1DM of long duration (>20 years). - Severe CKD (eGFR <30 mL/min/1.73 m^2^). - A calculated SCORE ≥10% for 10-year risk of fatal CVD. - FH with ASCVD or with another major risk factor. | People with:   - Markedly elevated single risk factors, in particular TC >8mmol/L (>310 mg/dL), LDL-C >4.9 mmol/L (>190 mg/dL), or BP ≥180/110 mmHg. - Patients with FH without other major risk factors. - Patients with DM without target organ damage^a^, with DM duration ≥10 years or another additional risk factor. - Moderate CKD (eGFR 30-59 mL/min/1.73 m^2^). - A calculated SCORE ≥5% and <10% for 10-year risk of fatal CVD. |

ASCVD = atherosclerotic cardiovascular disease; ACS = acute coronary syndrome; BP = blood pressure; CABG = coronary artery bypass graft surgery; CKD = chronic kidney disease; CT = computed tomography; CVD = cardiovascular disease; DM = diabetes mellitus; eGFR = estimated glomerular filtration rate; FH = familial hypercholesterolemia; LDL-C = low-density lipoprotein cholesterol; MI = myocardial infarction; PCI = percutaneous coronary intervention; SCORE = Systematic Coronary Risk Estimation; T1DM = type 1 DM; TC = total cholesterol; TIA = transient ischemic attack.

^a^ Target organ damage is defined as microalbuminuria, retinopathy, or neuropathy.

Source: Mach et al. (2020)[1]

***S2 - Literature review on relative risks***

We restricted our review to meta-analyses only, thus conducting an umbrella review. The literature search was carried out on January 29, 2021 using the MEDLINE database. We used the search terms (“low-density lipoprotein” [ti] OR “LDL” [ti] OR “cholesterol” [ti]) AND (“risk ratio” OR “hazard ratio” OR “relative risk”) AND (meta-analysis). The search resulted in 67 unique hits. We searched the references cited in the publications (“backward snowballing”) for studies with >50,000 observations that reported the relationship between cholesterol and cardiovascular diseases (ICD10 I00-I99). We identified four relevant studies (Asia Pacific Cohort Studies Collaboration, 2003; Kwon et al., 2019; Prospective Studies Collaboration, 2007; Singh et al., 2013) and finally decided to use results from the *Prospective Studies Collaboration* (2007) as a basis for our analysis, because it provided a comprehensive distinction between diseases, sex, and age groups.

***S3 - Validation of the prevalence estimate***

According to our estimation, the proportion of the population with total cholesterol levels above 7 mmol/l – and thus regarded to be affected by hypercholesterolemia – is around 1 % under the age of 30, around 4 % in women and around 9 % in men aged 30 to 44. The share is highest among 45- to 64-year-olds of both sexes with approximately 18 % each. Compared to the reported prevalence of hypercholesterolemia of the same study from which we obtained the means and standard deviations of cholesterol levels[2], we overestimate the shares by 3-5 percentage points (for men and women of different age groups). However, since the results of the NCD Risk Factor Collaboration[3] indicate that average total cholesterol levels in Austria are higher than in Germany, we conclude that a slight overestimation of the proportions in the high cholesterol groups might adequately represent the Austrian situation.

***S4 - Prevalence of hypercholesterolemia and prevalence of hypercholesterolemia with high and very high risk of cardiovascular disease***

Table A-1 Estimated prevalence of hypercholesterolemia, by age and risk groups

| Age | Hypercholesterolemia | Hypercholesterolemia & high risk | Hypercholesterolemia & very high risk |
| --- | --- | --- | --- |
| 15–19 | 29.7% | 29.7% | 0.0% |
| 20–24 | 29.7% | 29.7% | 0.0% |
| 25–29 | 29.7% | 29.7% | 0.0% |
| 30–34 | 47.7% | 47.7% | 0.0% |
| 35–39 | 47.7% | 47.7% | 0.0% |
| 40–44 | 47.8% | 47.3% | 0.5% |
| 45–49 | 60.0% | 58.9% | 1.1% |
| 50–54 | 61.4% | 59.5% | 1.9% |
| 55–59 | 64.8% | 60.6% | 4.2% |
| 60–64 | 66.8% | 61.3% | 5.5% |
| 65–69 | 75.5% | 64.3% | 11.2% |
| 70–74 | 79.7% | 65.8% | 13.9% |
| 75–79 | 88.9% | 69.1% | 19.8% |

Note: estimate based on results in Frick [4], Dippel et al. [5].

***S5 - Data regarding population, death rate, care allowances, sickness benefits, disability pensions, employment rates, and wage costs***

Austria’s population was 8.2 million in 2019. A total of 83,386 deaths were registered in 2019, 35 % of which were caused by cardiovascular diseases [6].

For 11.3 % of newly entitled beneficiaries of care allowance, cardiovascular diseases were registered as the cause for entitlement. In Austria, care allowances are paid out in seven levels according to care needs. The amount paid per month in 2019 was €157.30 for the first level, €290.00 for the second level, €451.80 for the third level, €677.60 for the fourth level, €920.30 for the fifth level, €1,285.20 for the sixth level and €1,688.90 for the seventh level. We received the respective data as well as data on disability pensions from the Umbrella Organization of Austrian Social Security Institutions.

Entitlement to disability pension arises if regular work cannot be carried out due to the state of health but retirement age has not been reached. Out of all newly entitled beneficiaries of disability pension in 2019, 5 % of women and 15 % of men were entitled due to cardiovascular diseases.

Around 5 % of total expenditure for sickness benefits and 3.8 % of sick leaves were attributable to cardiovascular diseases. Sickness benefits serve as a substitute for loss of earnings in the event of temporary incapacity to work because of an illness. We received data on sickness benefits and sick leaves by sex, age, and disease groups (ICD-10) from the Austrian Health Insurance Fund.

Austria’s labor force participation rate, necessary to calculate the indirect costs of hypercholesterolemia, was 74.2 % in 2019. Participation rates by sex and age groups as well as the respective total wage costs which we used in our analysis are given in tables A-5 and A-6.

Table A-5 Labor force participation rate by sex and age groups [7], 2019

| Age | Men | Women |
| --- | --- | --- |
| 15–19 | 38% | 25% |
| 20–24 | 68% | 59% |
| 25–29 | 84% | 76% |
| 30–34 | 91% | 78% |
| 35–39 | 93% | 82% |
| 40–44 | 92% | 85% |
| 45–49 | 90% | 86% |
| 50–54 | 89% | 85% |
| 55–59 | 84% | 74% |
| 60–64 | 45% | 15% |
| 65–69 | 3% | 1% |

Table A-6 Total annual labor costs, by sex and age groups [6], 2019

| Age | Men | Women |
| --- | --- | --- |
| 15–19 | 7,680 | 5,155 |
| 20–24 | 22,947 | 17,799 |
| 25–29 | 34,480 | 26,542 |
| 30–34 | 42,643 | 29,104 |
| 35–39 | 48,809 | 29,840 |
| 40–44 | 54,006 | 33,096 |
| 45–49 | 58,898 | 37,846 |
| 50–54 | 62,495 | 40,460 |
| 55–59 | 64,702 | 42,845 |
| 60–64 | 76,386 | 60,248 |
| 65–69 | 83,036 | 34,650 |
| 70–74 | 42 371 | 19 572 |

Note: Labor costs include employees’ and employers’ social security contributions and payroll taxes

***References***

1. Mach, F., Baigent, C., Catapano, A.L., Koskinas, K.C., Casula, M., Badimon, L., Chapman, M.J., De Backer, G.G., Delgado, V., Ference, B.A., Graham, I.M., Halliday, A., Landmesser, U., Mihaylova, B., Pedersen, T.R., Riccardi, G., Richter, D.J., Sabatine, M.S., Taskinen, M.-R., Tokgozoglu, L., Wiklund, O., ESC Scientific Document Group, Mueller, C., Drexel, H., Aboyans, V., Corsini, A., Doehner, W., Farnier, M., Gigante, B., Kayikcioglu, M., Krstacic, G., Lambrinou, E., Lewis, B.S., Masip, J., Moulin, P., Petersen, S., Petronio, A.S., Piepoli, M.F., Pintó, X., Räber, L., Ray, K.K., Reiner, Ž., Riesen, W.F., Roffi, M., Schmid, J.-P., Shlyakhto, E., Simpson, I.A., Stroes, E., Sudano, I., Tselepis, A.D., Viigimaa, M., Vindis, C., Vonbank, A., Vrablik, M., Vrsalovic, M., Zamorano, J.L., Collet, J.-P., Koskinas, K.C., Casula, M., Badimon, L., John Chapman, M., De Backer, G.G., Delgado, V., Ference, B.A., Graham, I.M., Halliday, A., Landmesser, U., Mihaylova, B., Pedersen, T.R., Riccardi, G., Richter, D.J., Sabatine, M.S., Taskinen, M.-R., Tokgozoglu, L., Wiklund, O., Windecker, S., Aboyans, V., Baigent, C., Collet, J.-P., Dean, V., Delgado, V., Fitzsimons, D., Gale, C.P., Grobbee, D., Halvorsen, S., Hindricks, G., Iung, B., Jüni, P., Katus, H.A., Landmesser, U., Leclercq, C., Lettino, M., Lewis, B.S., Merkely, B., Mueller, C., Petersen, S., Petronio, A.S., Richter, D.J., Roffi, M., Shlyakhto, E., Simpson, I.A., Sousa-Uva, M., Touyz, R.M., Nibouche, D., Zelveian, P.H., Siostrzonek, P., Najafov, R., van de Borne, P., Pojskic, B., Postadzhiyan, A., Kypris, L., Špinar, J., Larsen, M.L., Eldin, H.S., Viigimaa, M., Strandberg, T.E., Ferrières, J., Agladze, R., Laufs, U., Rallidis, L., Bajnok, L., Gudjónsson, T., Maher, V., Henkin, Y., Gulizia, M.M., Mussagaliyeva, A., Bajraktari, G., Kerimkulova, A., Latkovskis, G., Hamoui, O., Slapikas, R., Visser, L., Dingli, P., Ivanov, V., Boskovic, A., Nazzi, M., Visseren, F., Mitevska, I., Retterstøl, K., Jankowski, P., Fontes-Carvalho, R., Gaita, D., Ezhov, M., Foscoli, M., Giga, V., Pella, D., Fras, Z., de Isla, L.P., Hagström, E., Lehmann, R., Abid, L., Ozdogan, O., Mitchenko, O., Patel, R.S.: 2019 ESC/EAS Guidelines for the management of dyslipidaemias: lipid modification to reduce cardiovascular risk. Eur. Heart J. 41, 111–188 (2020). https://doi.org/10.1093/eurheartj/ehz455

2. Scheidt-Nave, C., Du, Y., Knopf, H., Schienkiewitz, A., Ziese, T., Nowossadeck, E., Gößwald, A., Busch, M.A.: Verbreitung von Fettstoffwechselstörungen bei Erwachsenen in Deutschland: Ergebnisse der Studie zur Gesundheit Erwachsener in Deutschland (DEGS1). Bundesgesundheitsblatt - Gesundheitsforschung - Gesundheitsschutz. 56, 661–667 (2013). https://doi.org/10.1007/s00103-013-1670-0

3. NCD Risk Factor Collaboration (NCD-RisC), Taddei, C., Zhou, B., Bixby, H., Carrillo-Larco, R.M., Danaei, G., Jackson, R.T., Farzadfar, F., Sophiea, M.K., Di Cesare, M., Iurilli, M.L.C., Martinez, A.R., Asghari, G., Dhana, K., Gulayin, P., Kakarmath, S., Santero, M., Voortman, T., Riley, L.M., Cowan, M.J., Savin, S., Bennett, J.E., Stevens, G.A., Paciorek, C.J., Aekplakorn, W., Cifkova, R., Giampaoli, S., Kengne, A.P., Khang, Y.-H., Kuulasmaa, K., Laxmaiah, A., Margozzini, P., Mathur, P., Nordestgaard, B.G., Zhao, D., Aadahl, M., Abarca-Gómez, L., Rahim, H.A., Abu-Rmeileh, N.M., Acosta-Cazares, B., Adams, R.J., Agdeppa, I.A., Aghazadeh-Attari, J., Aguilar-Salinas, C.A., Agyemang, C., Ahluwalia, T.S., Ahmad, N.A., Ahmadi, A., Ahmadi, N., Ahmed, S.H., Ahrens, W., Ajlouni, K., Alarouj, M., AlBuhairan, F., AlDhukair, S., Ali, M.M., Alkandari, A., Alkerwi, A., Aly, E., Amarapurkar, D.N., Amouyel, P., Andersen, L.B., Anderssen, S.A., Anjana, R.M., Ansari-Moghaddam, A., Aounallah-Skhiri, H., Araújo, J., Ariansen, I., Aris, T., Arku, R.E., Arlappa, N., Aryal, K.K., Aspelund, T., Assunção, M.C.F., Auvinen, J., Avdicová, M., Azevedo, A., Azizi, F., Azmin, M., Balakrishna, N., Bamoshmoosh, M., Banach, M., Bandosz, P., Banegas, J.R., Barbagallo, C.M., Barceló, A., Barkat, A., Bata, I., Batieha, A.M., Batyrbek, A., Baur, L.A., Beaglehole, R., Belavendra, A., Ben Romdhane, H., Benet, M., Benn, M., Berkinbayev, S., Bernabe-Ortiz, A., Bernotiene, G., Bettiol, H., Bhargava, S.K., Bi, Y., Bienek, A., Bikbov, M., Bista, B., Bjerregaard, P., Bjertness, E., Bjertness, M.B., Björkelund, C., Bloch, K.V., Blokstra, A., Bo, S., Boehm, B.O., Boggia, J.G., Boissonnet, C.P., Bonaccio, M., Bongard, V., Borchini, R., Borghs, H., Bovet, P., Brajkovich, I., Breckenkamp, J., Brenner, H., Brewster, L.M., Bruno, G., Bugge, A., Busch, M.A., de León, A.C., Cacciottolo, J., Can, G., Cândido, A.P.C., Capanzana, M.V., Capuano, E., Capuano, V., Cardoso, V.C., Carvalho, J., Casanueva, F.F., Censi, L., Chadjigeorgiou, C.A., Chamukuttan, S., Chaturvedi, N., Chen, C.-J., Chen, F., Chen, S., Cheng, C.-Y., Cheraghian, B., Chetrit, A., Chiou, S.-T., Chirlaque, M.-D., Cho, B., Cho, Y., Chudek, J., Claessens, F., Clarke, J., Clays, E., Concin, H., Confortin, S.C., Cooper, C., Costanzo, S., Cottel, D., Cowell, C., Crujeiras, A.B., Csilla, S., Cui, L., Cureau, F.V., D’Arrigo, G., d’Orsi, E., Dallongeville, J., Damasceno, A., Dankner, R., Dantoft, T.M., Dauchet, L., Davletov, K., De Backer, G., De Bacquer, D., de Gaetano, G., De Henauw, S., de Oliveira, P.D., De Ridder, D., De Smedt, D., Deepa, M., Deev, A.D., Dehghan, A., Delisle, H., Dennison, E., Deschamps, V., Dhimal, M., Di Castelnuovo, A.F., Dika, Z., Djalalinia, S., Dobson, A.J., Donfrancesco, C., Donoso, S.P., Döring, A., Dorobantu, M., Dragano, N., Drygas, W., Du, Y., Duante, C.A., Duda, R.B., Dzerve, V., Dziankowska-Zaborszczyk, E., Eddie, R., Eftekhar, E., Eggertsen, R., Eghtesad, S., Eiben, G., Ekelund, U., El Ati, J., Eldemire-Shearer, D., Eliasen, M., Elosua, R., Erasmus, R.T., Erbel, R., Erem, C., Eriksen, L., Eriksson, J.G., Escobedo-de la Peña, J., Eslami, S., Esmaeili, A., Evans, A., Faeh, D., Fall, C.H., Faramarzi, E., Farjam, M., Fattahi, M.R., Felix-Redondo, F.J., Ferguson, T.S., Fernández-Bergés, D., Ferrante, D., Ferrari, M., Ferreccio, C., Ferrieres, J., Föger, B., Foo, L.H., Forslund, A.-S., Forsner, M., Fouad, H.M., Francis, D.K., do Carmo Franco, M., Franco, O.H., Frontera, G., Fujita, Y., Fumihiko, M., Furusawa, T., Gaciong, Z., Galvano, F., Gao, J., Garcia-de-la-Hera, M., Garnett, S.P., Gaspoz, J.-M., Gasull, M., Gazzinelli, A., Geleijnse, J.M., Ghanbari, A., Ghasemi, E., Gheorghe-Fronea, O.-F., Ghimire, A., Gianfagna, F., Gill, T.K., Giovannelli, J., Gironella, G., Giwercman, A., Goltzman, D., Gonçalves, H., Gonzalez-Chica, D.A., Gonzalez-Gross, M., González-Rivas, J.P., González-Villalpando, C., González-Villalpando, M.-E., Gonzalez, A.R., Gottrand, F., Graff-Iversen, S., Grafnetter, D., Gregor, R.D., Grodzicki, T., Grøntved, A., Grosso, G., Gruden, G., Gu, D., Guallar-Castillón, P., Guan, O.P., Gudmundsson, E.F., Gudnason, V., Guerrero, R., Guessous, I., Gunnlaugsdottir, J., Gupta, R., Gutierrez, L., Gutzwiller, F., Ha, S., Hadaegh, F., Haghshenas, R., Hakimi, H., Hambleton, I.R., Hamzeh, B., Hantunen, S., Kumar, R.H., Hashemi-Shahri, S.M., Hata, J., Haugsgjerd, T., Hayes, A.J., He, J., He, Y., Hendriks, M.E., Henriques, A., Herrala, S., Heshmat, R., Hill, A.G., Ho, S.Y., Ho, S.C., Hobbs, M., Hofman, A., Homayounfar, R., Hopman, W.M., Horimoto, A.R.V.R., Hormiga, C.M., Horta, B.L., Houti, L., Howitt, C., Htay, T.T., Htet, A.S., Htike, M.M.T., Huerta, J.M., Huhtaniemi, I.T., Huisman, M., Hunsberger, M.L., Husseini, A.S., Huybrechts, I., Hwalla, N., Iacoviello, L., Iannone, A.G., Ibrahim, M.M., Wong, N.I., Iglesia, I., Ikeda, N., Ikram, M.A., Iotova, V., Irazola, V.E., Ishida, T., Islam, M., al-Safi Ismail, A., Iwasaki, M., Jacobs, J.M., Jaddou, H.Y., Jafar, T., James, K., Jamrozik, K., Janszky, I., Janus, E., Jarvelin, M.-R., Jasienska, G., Jelakovic, A., Jelakovic, B., Jennings, G., Jensen, G.B., Jeong, S., Jha, A.K., Jiang, C.Q., Jimenez, R.O., Jöckel, K.-H., Joffres, M., Jokelainen, J.J., Jonas, J.B., Jørgensen, T., Joshi, P., Joukar, F., Józwiak, J., Juolevi, A., Kafatos, A., Kajantie, E.O., Kalter-Leibovici, O., Kamaruddin, N.A., Kamstrup, P.R., Karki, K.B., Katz, J., Kauhanen, J., Kaur, P., Kavousi, M., Kazakbaeva, G., Keil, U., Keinänen-Kiukaanniemi, S., Kelishadi, R., Keramati, M., Kerimkulova, A., Kersting, M., Khader, Y.S., Khalili, D., Khateeb, M., Kheradmand, M., Khosravi, A., Kiechl-Kohlendorfer, U., Kiechl, S., Killewo, J., Kim, H.C., Kim, J., Kim, Y.-Y., Klumbiene, J., Knoflach, M., Ko, S., Kohler, H.-P., Kohler, I.V., Kolle, E., Kolsteren, P., König, J., Korpelainen, R., Korrovits, P., Kos, J., Koskinen, S., Kouda, K., Kowlessur, S., Kratzer, W., Kriemler, S., Kristensen, P.L., Krokstad, S., Kromhout, D., Kujala, U.M., Kurjata, P., Kyobutungi, C., Laamiri, F.Z., Laatikainen, T., Lachat, C., Laid, Y., Lam, T.H., Lambrinou, C.-P., Lanska, V., Lappas, G., Larijani, B., Latt, T.S., Laugsand, L.E., Lazo-Porras, M., Lee, J., Lee, J., Lehmann, N., Lehtimäki, T., Levitt, N.S., Li, Y., Lilly, C.L., Lim, W.-Y., Lima-Costa, M.F., Lin, H.-H., Lin, X., Lin, Y.-T., Lind, L., Linneberg, A., Lissner, L., Liu, J., Loit, H.-M., Lopez-Garcia, E., Lopez, T., Lotufo, P.A., Lozano, J.E., Luksiene, D., Lundqvist, A., Lundqvist, R., Lunet, N., Ma, G., Machado-Coelho, G.L.L., Machado-Rodrigues, A.M., Machi, S., Madar, A.A., Maggi, S., Magliano, D.J., Magriplis, E., Mahasampath, G., Maire, B., Makdisse, M., Malekzadeh, F., Malekzadeh, R., Rao, K.M., Manios, Y., Mann, J.I., Mansour-Ghanaei, F., Manzato, E., Marques-Vidal, P., Martorell, R., Mascarenhas, L.P., Mathiesen, E.B., Matsha, T.E., Mavrogianni, C., McFarlane, S.R., McGarvey, S.T., McLachlan, S., McLean, R.M., McLean, S.B., McNulty, B.A., Mediene-Benchekor, S., Mehdipour, P., Mehlig, K., Mehrparvar, A.H., Meirhaeghe, A., Meisinger, C., Menezes, A.M.B., Menon, G.R., Merat, S., Mereke, A., Meshram, I.I., Metcalf, P., Meyer, H.E., Mi, J., Michels, N., Miller, J.C., Minderico, C.S., Mini, G.K., Miquel, J.F., Miranda, J.J., Mirjalili, M.R., Mirrakhimov, E., Modesti, P.A., Moghaddam, S.S., Mohajer, B., Mohamed, M.K., Mohammad, K., Mohammadi, Z., Mohammadifard, N., Mohammadpourhodki, R., Mohan, V., Mohanna, S., Yusoff, M.F.M., Mohebbi, I., Mohebi, F., Moitry, M., Møllehave, L.T., Møller, N.C., Molnár, D., Momenan, A., Mondo, C.K., Monterrubio-Flores, E., Moosazadeh, M., Morejon, A., Moreno, L.A., Morgan, K., Morin, S.N., Moschonis, G., Mossakowska, M., Mostafa, A., Mota, J., Motlagh, M.E., Motta, J., Msyamboza, K.P., Muiesan, M.L., Müller-Nurasyid, M., Mursu, J., Mustafa, N., Nabipour, I., Naderimagham, S., Nagel, G., Naidu, B.M., Najafi, F., Nakamura, H., Námešná, J., Nang, E.E.K., Nangia, V.B., Nauck, M., Neal, W.A., Nejatizadeh, A., Nenko, I., Nervi, F., Nguyen, N.D., Nguyen, Q.N., Nieto-Martínez, R.E., Nihal, T., Niiranen, T.J., Ning, G., Ninomiya, T., Noale, M., Noboa, O.A., Noto, D., Nsour, M.A., Nuhoğlu, I., O’Neill, T.W., O’Reilly, D., Ochoa-Avilés, A.M., Oh, K., Ohtsuka, R., Olafsson, Ö., Olié, V., Oliveira, I.O., Omar, M.A., Onat, A., Ong, S.K., Ordunez, P., Ornelas, R., Ortiz, P.J., Osmond, C., Ostojic, S.M., Ostovar, A., Otero, J.A., Owusu-Dabo, E., Paccaud, F.M., Pahomova, E., Pajak, A., Palmieri, L., Pan, W.-H., Panda-Jonas, S., Panza, F., Parnell, W.R., Patel, N.D., Peer, N., Peixoto, S.V., Peltonen, M., Pereira, A.C., Peters, A., Petersmann, A., Petkeviciene, J., Peykari, N., Pham, S.T., Pichardo, R.N., Pigeot, I., Pilav, A., Pilotto, L., Piwonska, A., Pizarro, A.N., Plans-Rubió, P., Plata, S., Pohlabeln, H., Porta, M., Portegies, M.L.P., Poudyal, A., Pourfarzi, F., Poustchi, H., Pradeepa, R., Price, J.F., Providencia, R., Puder, J.J., Puhakka, S.E., Punab, M., Qorbani, M., Bao, T.Q., Radisauskas, R., Rahimikazerooni, S., Raitakari, O., Rao, S.R., Ramachandran, A., Ramos, E., Ramos, R., Rampal, L., Rampal, S., Redon, J., Reganit, P.F.M., Revilla, L., Rezaianzadeh, A., Ribeiro, R., Richter, A., Rigo, F., Rinke de Wit, T.F., Rodríguez-Artalejo, F., del Cristo Rodriguez-Perez, M., Rodríguez-Villamizar, L.A., Roggenbuck, U., Rojas-Martinez, R., Romaguera, D., Romeo, E.L., Rosengren, A., Roy, J.G.R., Rubinstein, A., Ruidavets, J.-B., Ruiz-Betancourt, B.S., Russo, P., Rust, P., Rutkowski, M., Sabanayagam, C., Sachdev, H.S., Sadjadi, A., Safarpour, A.R., Safiri, S., Saidi, O., Saki, N., Salanave, B., Salmerón, D., Salomaa, V., Salonen, J.T., Salvetti, M., Sánchez-Abanto, J., Sans, S., Santaliestra-Pasías, A.M., Santos, D.A., Santos, M.P., Santos, R., Saramies, J.L., Sardinha, L.B., Sarrafzadegan, N., Saum, K.-U., Savva, S.C., Sawada, N., Sbaraini, M., Scazufca, M., Schaan, B.D., Schargrodsky, H., Scheidt-Nave, C., Schienkiewitz, A., Schipf, S., Schmidt, C.O., Schöttker, B., Schramm, S., Sebert, S., Sein, A.A., Sen, A., Sepanlou, S.G., Servais, J., Shakeri, R., Shalnova, S.A., Shamah-Levy, T., Sharafkhah, M., Sharma, S.K., Shaw, J.E., Shayanrad, A., Shi, Z., Shibuya, K., Shimizu-Furusawa, H., Shin, D.W., Shin, Y., Shirani, M., Shiri, R., Shrestha, N., Si-Ramlee, K., Siani, A., Siantar, R., Sibai, A.M., Silva, D.A.S., Simon, M., Simons, J., Simons, L.A., Sjöström, M., Skaaby, T., Slowikowska-Hilczer, J., Slusarczyk, P., Smeeth, L., Snijder, M.B., Söderberg, S., Soemantri, A., Sofat, R., Solfrizzi, V., Somi, M.H., Sonestedt, E., Sørensen, T.I.A., Jérome, C.S., Soumaré, A., Sozmen, K., Sparrenberger, K., Staessen, J.A., Stathopoulou, M.G., Stavreski, B., Steene-Johannessen, J., Stehle, P., Stein, A.D., Stessman, J., Stevanović, R., Stieber, J., Stöckl, D., Stokwiszewski, J., Stronks, K., Strufaldi, M.W., Suárez-Medina, R., Sun, C.-A., Sundström, J., Suriyawongpaisal, P., Sy, R.G., Sylva, R.C., Szklo, M., Tai, E.S., Tamosiunas, A., Tan, E.J., Tarawneh, M.R., Tarqui-Mamani, C.B., Taylor, A., Taylor, J., Tell, G.S., Tello, T., Thankappan, K.R., Thijs, L., Thuesen, B.H., Toft, U., Tolonen, H.K., Tolstrup, J.S., Topbas, M., Topór-Madry, R., Tormo, M.J., Tornaritis, M.J., Torrent, M., Torres-Collado, L., Traissac, P., Trinh, O.T.H., Truthmann, J., Tsugane, S., Tulloch-Reid, M.K., Tuomainen, T.-P., Tuomilehto, J., Tybjaerg-Hansen, A., Tzourio, C., Ueda, P., Ugel, E., Ulmer, H., Unal, B., Uusitalo, H.M.T., Valdivia, G., Valvi, D., van Dam, R.M., van der Schouw, Y.T., Van Herck, K., Van Minh, H., van Rossem, L., Van Schoor, N.M., van Valkengoed, I.G.M., Vanderschueren, D., Vanuzzo, D., Varbo, A., Varona-Pérez, P., Vasan, S.K., Vatten, L., Vega, T., Veidebaum, T., Velasquez-Melendez, G., Venero-Fernández, S.J., Veronesi, G., Verschuren, W.M.M., Victora, C.G., Vidiawati, D., Viet, L., Villalpando, S., Vioque, J., Virtanen, J.K., Visvikis-Siest, S., Viswanathan, B., Vlasoff, T., Vollenweider, P., Voutilainen, A., Wade, A.N., Wagner, A., Walton, J., Bebakar, W.M.W., Mohamud, W.N.W., Wang, M.-D., Wang, N., Wang, Q., Wang, Y.X., Wang, Y.-W., Wannamethee, S.G., Wedderkopp, N., Wei, W., Whincup, P.H., Widhalm, K., Widyahening, I.S., Wiecek, A., Wijga, A.H., Wilks, R.J., Willeit, J., Willeit, P., Wilsgaard, T., Wojtyniak, B., Wong-McClure, R.A., Wong, A., Wong, T.Y., Woo, J., Woodward, M., Wu, F.C., Wu, S., Xu, H., Xu, L., Yan, W., Yang, X., Yasuharu, T., Ye, X., Yeow, T.P., Yiallouros, P.K., Yoosefi, M., Yoshihara, A., You, S.-L., Younger-Coleman, N.O., Yusoff, A.F., Zainuddin, A.A., Zakavi, S.R., Zali, M.R., Zamani, F., Zambon, S., Zampelas, A., Zaw, K.K., Zdrojewski, T., Vrkic, T.Z., Zhang, Z.-Y., Zhao, W., Zhen, S., Zheng, Y., Zholdin, B., Zhussupov, B., Zoghlami, N., Cisneros, J.Z., Gregg, E.W., Ezzati, M.: Repositioning of the global epicentre of non-optimal cholesterol. Nature. 582, 73–77 (2020). https://doi.org/10.1038/s41586-020-2338-1

4. Frick, U., Rehm, J.: Konsistente Schätzung von Prävalenz, Inzidenz und krankheitsspezifischer Mortalität für Öster-reich: Koronare Herzkrankheiten (ICD10: I20 bis I25). Hauptverband. (2011)

5. Dippel, F.-W., Parhofer, K., Müller-Bohn, T., Gebhardt, S., Kostev, K.: Sekundärdatenanalyse zur Ermittlung der Prävalenz von kardiovaskulären Hochrisiko-Patienten mit Hypercholesterinämie und therapierefraktärem Behandlungsverlauf. Dtsch. Med. Wochenschr. 142, e34–e41 (2017). https://doi.org/10.1055/s-0042-123977

6. Statistik Austria: STATcube – Statistische Datenbank., http://www.statistik.at/web_de/services/statcube/index.html

7. AMS: Arbeitsmarktlage 2019: Erwerbsquoten nach Geschlecht und Alter, Jahresdurchschnitt. (2021)
